# The Role of Community Health Workers in Improving Diabetes Device Use Among Youth With Type 1 Diabetes: A Web-Based Qualitative Study Using Human-Centered Design With Clinicians

**DOI:** 10.2196/76387

**Published:** 2025-08-28

**Authors:** Charlotte Wang Chen, Alexa Jacqueline Durante, Margaret Grace Maynard, Marina Reznik, Lori Laffel, Shivani Agarwal

**Affiliations:** 1 Department of Endocrinology Children's Hospital at Montefiore Einstein Bronx United States; 2 Albert Einstein College of Medicine Bronx, NY United States; 3 Children's Hospital at Montefiore Einstein Bronx, NY United States; 4 Joslin Diabetes Center Boston, MA United States; 5 Fleischer Institute of Diabetes and Metabolism Montefiore-Einstein Bronx, NY United States

**Keywords:** diabetes management, diabetes technology, health disparities, type 1 diabetes

## Abstract

**Background:**

Inequity in diabetes technology use persists among Black and Hispanic youth with type 1 diabetes (T1D). Community health workers (CHWs) can address social and clinical barriers to diabetes device use. However, more information is needed on clinicians’ perceptions to inform the development of a CHW model for youth with T1D.

**Objective:**

This study aimed to identify barriers to diabetes technology use and cocreate solutions in collaboration with diabetes and school-based clinicians serving Black and Hispanic youth with T1D.

**Methods:**

Using human-centered design, the study team conducted 2-hour web-based workshops with clinicians from a diabetes clinic or school-based clinics at a safety net hospital in the Bronx, New York. The workshops promoted active ideation of barriers and co-design of a CHW intervention prototype to address self-reported challenges. Workshops were analyzed using a qualitative inductive approach.

**Results:**

A total of 17 participants completed the human-centered design workshops and surveys. Of these, 11 (65%) were clinicians from the diabetes clinic and 6 (35%) were school-based clinicians from elementary, middle, and high schools in the Bronx. A total of 4 workshops were conducted. The perceived diabetes device barriers for youth with T1D and their families by participants were general health-related social needs (HRSNs) and diabetes technology–specific HRSNs that interfered with technology uptake, such as housing and financial insecurity, as well as digital social needs; and difficulty navigating health care systems, insurance, and pharmacy benefits due to the high level of care coordination required by caregivers. In addition, the participants identified barriers that interfered with their ability to support youth with T1D with diabetes technology, such as limited support for using diabetes technology in school and lack of time and technology support to troubleshoot problems in diabetes clinics. Ways in which a CHW could help mitigate these barriers include (1) identifying and addressing HRSNs by directing patients to appropriate resources; (2) providing peer support for caregivers to navigate diabetes device logistics; (3) acting as a school liaison to improve communication and coordination between caregivers, schools, and diabetes clinicians; and (4) offering administrative support to offload the logistical burden of clinicians.

**Conclusions:**

Important needs related to specialized technology support, enhanced care coordination, family-clinician communication, and administrative task shifting were identified by clinicians to inform a CHW model for youth with T1D. Continued co-design and pilot testing are needed to refine the model.

## Introduction

### Background

Type 1 diabetes (T1D) is one of the most common chronic illnesses in youth, impacting 304,000 children and adolescents aged <20 years in the United States, most of whom spend the majority of their time in schools [1]. Managing T1D is particularly challenging, as it involves carbohydrate counting, insulin dosage calculations, frequent glucose monitoring, and attention to physical activity [2]. As a result, it is not surprising that 82% of youth with T1D do not achieve the glycemic target of hemoglobin A_1c_ of <7% recommended by national and international guidelines [3-5]. In addition, significant inequity exists in glycemic outcomes among different demographic groups, with non-Hispanic White youth having 1.34 greater odds of achieving hemoglobin A_1c_ <7% compared with Black and Hispanic youth [6].

Diabetes technologies such as insulin pumps, continuous glucose monitors, and automated insulin delivery systems can reduce the burden associated with diabetes management and improve the quality of life in youth with T1D [7-11]. However, Black and Hispanic youth with T1D are less likely to initiate and use them compared with non-Hispanic White youth with T1D, regardless of socioeconomic status [12-15]. Increasing diabetes device use in this population is a potential mechanism to reduce inequity in health outcomes.

Numerous studies have explored social-ecological factors that contribute to lower device uptake among Black and Hispanic youth with T1D [16-20]. Key findings include the impact of longstanding structural inequities, structural racism, physician bias, unmet social needs, barriers at school, and challenges related to care coordination [6,21-23]. Recognizing that these barriers exist at individual, family, and community levels, community-based interventions can be used to address these barriers in the environments where Black and Hispanic youth with T1D spend the majority of their time, such as school settings.

A community health worker (CHW) model is a potential community-based intervention that can address inequity associated with diabetes technology use. CHWs are trusted community members who can provide various services, ranging from basic patient education to social needs support to linking different care sites, such as schools and clinics [24-28]. For example, previous CHW interventions for youth with asthma have demonstrated significant reductions in acute care use, inpatient hospitalizations, days with symptoms, missed school days for children, and missed workdays for caregivers [24,27,29-31]. The success of the CHW model in other chronic childhood conditions suggests that a CHW care model can potentially improve diabetes device uptake and health outcomes in Black and Hispanic youth with T1D.

### Objectives

However, to actualize such a model for youth with T1D, it is necessary to understand how the CHW specialty care model can address specific barriers related to diabetes technology for clinicians, particularly in the clinic and school settings. Garnering perspectives from multidisciplinary clinicians who provide health care to these children in those settings could help fill this gap in the literature. Guided by human-centered design [32] and diabetes technology journey framework [33], this study aimed (1) to identify diabetes and school clinicians’ perspectives on barriers to diabetes device use among Black and Hispanic youth with T1D and (2) to help design a prototype of a CHW intervention to address these challenges.

## Methods

### Design

Given that the study aims to explore ways a CHW can address diabetes device barriers at diabetes and school-based clinics, the study was performed from a pragmatist point of view using a general inductive approach. The Standards for Reporting Qualitative Research checklist for qualitative research reporting was used for this report.

### Participants and Setting

The Children’s Hospital at Montefiore Einstein (CHAM) is a safety-net hospital in the Bronx, New York, part of the Montefiore Health System. The demographics of patients receiving care at CHAM consist of approximately 30% Black and 55% Hispanic, with most of the patients on public insurance. The New York Medicaid plan covers the majority of the costs associated with diabetes technology. The Montefiore School Health Program (MSHP), a branch of the Montefiore Health System, is the most extensive school-based health program in the United States [34]. MSHP provides coordinated primary care and preventative services (eg, physical examinations, immunizations, dental care, and mental health) as well as urgent care and referrals to subspecialists for school-aged children and adolescents in the Bronx [34]. School-based health programs in New York City have been shown to reduce school absences, parents’ time away from work, and hospital visits [35]. Services through the program are performed by physicians, nurse practitioners (NPs), and physician assistants.

Participants were diabetes clinicians employed at CHAM diabetes center (physicians, NPs, certified diabetes care and education specialists, social workers, and psychologists) or clinicians from the MSHP who provide care to Black or Hispanic youth with T1D aged 2-17 years. A staff member from MSHP helped identify potential MSHP clinicians, and CWC identified potential CHAM diabetes clinicians. Participants were recruited via flyers, emails, and word of mouth. Convenience sampling was used. Individuals interested in the study contacted the study coordinator (MGM). Participants were recruited from CHAM or MSHP in person at the clinic or over the phone.

### The Research Team

The research team included an early-career pediatric endocrinologist at CHAM (CWC), a senior pediatric endocrinologist (LL), a pediatric endocrinology fellow at CHAM (AJD), a study coordinator at CHAM (MGM), a general pediatrician (MR), and an adult endocrinologist (SA). The research team had extensive experience in all aspects of the study, including human-centered design (CWC, AJD, and SA), qualitative research (CWC, AJD, LL, MGM, MR, and SA), health services research (CWC, LL, MR, and SA), school-based interventions (CWC and MR), and type 1 clinical diabetes research (CWC, AJD, LL, and SA). A few study team members (CWC and AJD) work closely with clinicians from CHAM; however, the research team did not have relationships with clinicians from MSHP. Three study team members performed the workshops, with each workshop including 2 of the 3 personnel (CWC, AJD, and MGM).

### Human-Centered Design Web-Based Workshops

The multidisciplinary research team created the web-based interactive workshop using literature review, advisory boards, and clinical expertise. The human-centered design framework helped inform the workshops. Human-centered design relies on 3 steps: (1) understanding barriers from the target users; (2) analyzing and synthesizing to identify the main obstacles related to the issues; and (3) discussing and co-designing solutions with other attendees to the identified challenges, with specific details discussed such as who would deliver the intervention, when, to whom, and how [32]. This approach has been used previously to elicit perspectives from young adults with T1D [33] and caregivers of youth with T1D [23] on diabetes technology barriers and to cocreate solutions to address these barriers. The workshop materials were created over 2 months and practiced internally through mock sessions before deployment. These sessions helped to provide additional feedback, polish the materials, and effectively deliver the content.

The workshop was divided into 2 main activities. During the first activity, participants were introduced to the diabetes technology journey framework, which is defined as three stages: (1) learning and deciding to get technology; (2) getting and starting to use the technology; and (3) managing problems with technology [33]. Participants were asked to share barriers associated with each step of this process from their perspectives and their patients’ experiences.

In activity 2, participants prototyped the CHW intervention to address HRSNs and to support families and clinicians with the challenges identified in the first activity. They were asked to brainstorm specific ways that a CHW could address barriers affecting families of youth with T1D, as well as their barriers to prescribing diabetes devices and supporting families with device use. The participants’ real-time responses were shared on the screen to facilitate discussion. The interview guide for activities 1 and 2 from the workshop is shown in Table 1 and Textbox 1.

**Table 1:** Participant interview guide for activity 1.

Stages	Main questions	Prompting questions
Stage 1: learning about and deciding to get technology	What things have parents/guardians shared with you that make caring for diabetes hard? What has been your experience with diabetes technology so far? What are things that helped with learning about and deciding to get/prescribe diabetes technology? What got in the way of learning about and prescribing diabetes technology?	How did you learn about technology? What resources were provided to patients to learn about tech? How could you help patients navigate pros/cons of diabetes technology? How do you decide to prescribe tech?
Stage 2: getting and starting to use technology	What are things that helped you/your patients get and start using diabetes technology?	How did patients learn to manage diabetes in a new way? How did you learn how to use diabetes technology?
Stage 3: managing problems and situations with technology	What are things that helped you/your patients manage and troubleshoot problems after starting diabetes technology? What are things that got in the way of managing and troubleshooting problems after starting diabetes technology?	How do you figure out technical problems? How do you manage social situations? How do your patients decide to stop or keep diabetes tech? How has technology changed diabetes care?

**Participant interview guide for activity 2.**

**Activity 2: participant perceptions of a community health worker (CHW)**

- What is your perception of a CHW?
- How can a CHW help with social needs?
- How can a CHW help families on their diabetes technology journey?

### Procedure

Workshops were held on a privacy-protected video platform and video-recorded for analysis purposes. Baseline data on practice location, duration of practice, and demographics (credentials, practice location, and years of practice) were collected using REDCap (Research Electronic Data Capture; Vanderblit University) [36,37] questionnaires completed by participants.

### Ethical Considerations

This exploratory study was approved by the Institutional Review Board of the Albert Einstein College of Medicine (2022-14609). Participants were provided with information about the study. All participants provided written or oral informed consent per institutional review board approval. All study data have been deidentified to protect the privacy and confidentiality of the participants. Sensitive content was redacted from the transcripts to avoid identification. Participants who completed the 2-hour workshop and survey were each provided with a US $100 gift card as compensation.

### Qualitative Analysis

Workshops were recorded, transcribed, and coded in NVivo [38]. Three coders (CWC, AJD, and MGM) analyzed the workshops using a general inductive approach [39]. In activity 1, a coding structure with categories for barriers to diabetes technology use was created. In activity 2, categories for solutions using the CHW intervention were generated. The coding framework was updated with emergent codes, and codes were unified according to similarities. Overlapping concepts were developed into themes, with workshops continuing until thematic saturation was reached [40]. Information was obtained directly from clinicians who interact frequently with Black and Hispanic youth with T1D and their caregivers to ensure credibility. A senior team member reviewed the themes created by the 3 main coders, providing dependability and ensuring the methodology was followed appropriately. An audit trail was kept to track coding decisions.

### Quantitative Analysis

Results from the self-reported questionnaire were analyzed in Microsoft Excel and are presented as the mean (SD) for continuous variables. For categorical variables, frequencies or proportions are reported.

## Results

### Overview

Overall, 17 participants completed the human-centered design workshops and surveys. Of these, 11 (65%) were clinicians from the diabetes clinic and 6 (35%) were school-based clinicians from elementary, middle, and high schools in the Bronx. Descriptive data for participating clinicians are presented in Table 2. A total of 4 workshops, 3 groups with participants from the diabetes clinics and 1 with school-based clinicians, were conducted between September 2023 and December 2023. Each group had 3-6 participants.

**Figure 1:**
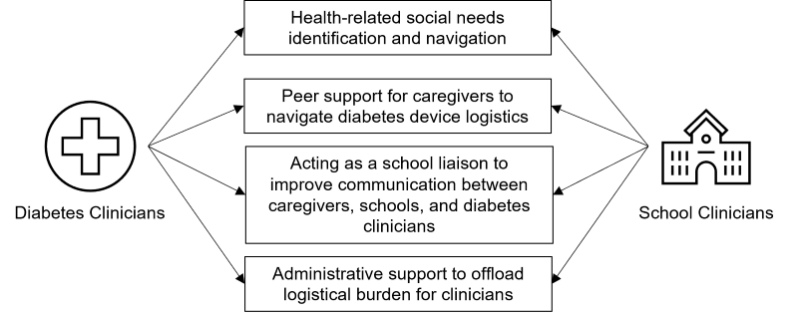
Cocreated solutions from the human-centered design workshops with diabetes and school clinicians.

In total, 4 key barriers and their corresponding solution themes were identified. Figure 1 represents the cocreated solutions by the clinicians.

**Table 2:** Participant demographics (N=17).

Demographics			Participants
**Participant clinical role, n (%)**			
	Physician	9 (53)	
	Nurse practitioner	6 (35)	
	Psychologist	1 (6)	
	Social worker	1 (6)	
**Practice location, n (%)**			
	Clinic	11 (65)	
	School	6 (35)	
Licensed CDECS^a^, n (%)			2 (12)
Practice duration (y), mean (SD; range)			10.8 (9.3; 2-34)

^a^CDECS: certified diabetes care and education specialist.

Clinicians identified two key barriers to the diabetes technology uptake and use for youth with T1D: (1) general and specific HRSNs interfering with diabetes technology; and (2) difficulty navigating health care systems, insurance, and pharmacy benefits for youth and their families. In addition, clinicians identified barriers that interfered with supporting their patients with diabetes technology, including limited technology support and infrastructure to support diabetes technology use in school and lack of technology support and time to troubleshoot diabetes devices in diabetes clinics.

### Caregiver-Centered Barriers and Solutions From the Clinicians’ Perspectives

#### Barrier 1: General and Specific HRSNs That Interfered With Diabetes Technology Uptake

Most participants (12/17, 71%) recognized that general HRSNs such as housing and financial insecurity presented a major barrier to diabetes device use for youth and their families. For diabetes technology–specific HRSNs, participants (5/17, 29%) acknowledged that digital social needs, such as not having access to a compatible smartphone, impacted whether youth with T1D received the full benefit of the automated insulin delivery system.

When we did telehealth, it was like a huge wow! We’re seeing into their homes for 30 min or an hour.... That was a big deal, seeing where people were living.... No wonder... they don’t care about diabetes.... They’re all sleeping on one mattress... that’s an issue.

Diabetes clinician #1

They had to pay out of pocket for... some supplies and insulin, so the mother would ration the insulin...so there were times where he wouldn’t come with his pump at all...Nothing would be connected, and...we wouldn’t find out till 12-1 o’clock and his sugar be unreadably high.

School clinician #4

A lot of the Android phones aren’t compatible with some CGMs [continuous glucose monitors], like even the newer ones.... And that can play actually into socioeconomics... because the Androids are cheaper than iPhones.

Diabetes clinician #7

#### Solution 1: HRSN Identification and Navigation

When presented with the general CHW model, participants (7/17, 41%) identified how this additional person could ease the diabetes technology journey for caregivers by assisting those with social needs.

There is really a need for... a one-on-one connection with someone who is linked to both the medical side and the community side. Because you can just... bang your head against the wall... feeling like you’re trying to support people, but they’re just running into barrier after barrier... and I can’t imagine how they feel, you know.

School clinician #1

Just being a resource. A lot of times people don’t access services because they’re not aware that [services] are available.

School clinician #2

A [CHW] could help direct families on a place to get [the correct type of smartphone] at... low cost or just help them navigate which ones would work [with diabetes devices].

Diabetes clinician #2

#### Barrier 2: Difficulty Navigating Health Care Systems, Insurance, and Pharmacy Benefits

Participants recognized families’ challenges with maintaining adequate device supplies (7/17, 41%). Participants from diabetes clinics emphasized the high level of organization that T1D requires from caregivers. Some participants (9/17, 53%) felt that certain families lacked a sense of empowerment or self-efficacy to manage the responsibilities associated with device use.

Not knowing what to do if they run out of supplies or knowing how... to manage supplies in general and stuff... that’s the biggest issue with technology or diabetes supplies.... They don’t understand how to use the system to get supplies...

Diabetes clinician #1

Participants (8/17, 47%) described families struggling with care coordination, which hindered the family’s ability to facilitate consistent technology use.

Some families are very good about calling in and going over those problems. I think other families kind of have, like a defeatist attitude. And they’re like, they can’t figure it out...and they just give up and stop working with the technology.

Diabetes clinician #3

Communication with the pharmacy, because... when they need another refill... the pharmacy, doesn’t just automatically send that out. They require that the parent call and sometimes there’s confusion if there’s... a prior authorization needed, and it needs to be renewed. Some parents are not as comfortable... reaching out to the pharmacy...

Diabetes clinician #4

#### Solution 2: Peer Support for Caregivers to Navigate Diabetes Device Logistics

To address challenges related to maintaining diabetes supplies and health care navigation, most participants (15/17, 88%) thought enhanced care coordination was needed to target issues families experience with pharmacies, insurance, and supply management.

Diabetes clinicians proposed having a “diabetes coach” to review technology education, keep track of appointments and device refills, and empower families to call the clinic, pharmacy, and diabetes device and insurance companies promptly (5/17, 29% overall and 45% of diabetes clinicians).

Helping families... make schedules... to like organize medications, know when things are expiring... For the disorganized families who don’t pay attention to when refills are due, like helping them kind of develop a strategy so that things don’t expire, and that they’re not waiting to the last minute for refills.

Diabetes clinician #3

Calling with the families..., being supportive and allowing the parent to make the phone call but being there with them to help support that.

Diabetes clinician #4

It’s the notion of catching things when they’re a small problem before it becomes a big problem.... If you have someone that you can go to, who you feel...you can share and can help to advocate...prevent some major catastrophes.

School clinician #3

### Clinician-Centered Barriers and Solutions

#### Barrier 3: Limited Support to Use Diabetes Technology in School

Participants from the diabetes clinic (5/17, 29% overall and 5/11, 45% of diabetes clinicians) felt burdened with the responsibility of responding to calls from school personnel regarding troubleshooting diabetes devices, educating school personnel on diabetes technology, and completing the required paperwork for diabetes management in school.

I still get a lot of calls, because the [school] nurse may not really understand how to use the technology. And, you know they don’t feel comfortable...they’re not touching the [CGM] or anything.... For some patients that I’ve worked with has resulted in... missing class, because maybe the monitor is going off or, for example, the pump, if the blood sugar is high...

Diabetes clinician #4

There’s a patient who...it’s very frustrating for the parent in terms of what’s going on at school...So I think that’s a barrier in terms of... miscommunication between school nurses and how to manage diabetes.... And then the burden falls on the parent because they call this parent frequently...

Diabetes clinician #5

School-based clinicians reported feeling overwhelmed by the wide varieties of diabetes technologies encountered in school clinics and expressed a lack of familiarity with all available devices (6/17, 35% overall and 6/6, 100% of school-based clinicians). In addition, the school-based clinicians (3/17, 18% overall and 3/6, 50% of school-based clinicians) emphasized the disruptions these diabetes technology malfunctions caused for the student and school-based clinics.

My patient has [a CGM] but it doesn’t talk to her insulin pump and I... have trouble wrapping my brain around that because I know that there’s... closed loop circuits.... One of the things that gets in the way of learning about tech is...there are so many options out there in the market. There’s not just like A-B-C, there’s just many, many things, and it’s always evolving and changing.

School clinician #4

#### Solution 3: Acting as a School Liaison to Improve Communication Between Caregivers, School, and Diabetes Clinicians

Some diabetes clinicians (3/17, 18% overall and 3/11, 27% of diabetes clinicians) endorsed redistributing some of the administrative work associated with diabetes management at school to a CHW. In addition, participants mentioned that CHWs could also help educate caregivers about their rights at school.

Helping parents ask for those school medical accommodation (504) meetings. I don’t think parents necessarily understand that it’s their right to ask for those things even though we try to tell them...

Diabetes clinician #3

On their part, school-based clinicians (4/17, 24% overall and 4/6, 67% of school-based clinicians) were enthusiastic about the potential to collaborate with CHWs, and they (6/17, 35% overall and 6/6, 100% of school-based clinicians) expressed a desire for additional training, especially on their students’ specific diabetes devices.

Let’s just say [the CHW] came to the school and educated. It would be a little bit more personalized and tailored to maybe that kid’s specific pump because there’s so many pumps.... My head spins when they’re educating us about a million pumps, so it would be nice if they were able to come to the school.

School clinician #4

I think that just being a liaison between a parent and whatever they’re trying to do, whether it’s pharmacy,... their doctor,... their school... just helping to get that access point.

Diabetes clinician #1

#### Barrier 4: Lack of Time and Technology Support to Troubleshoot Problems in Clinic

Most diabetes clinicians endorsed difficulties troubleshooting diabetes technology and keeping updated with technological advances (4/17, 24% overall and 4/11, 36% among diabetes clinicians). In addition, participants expressed frustration over the time-intensive nature of discussing and troubleshooting diabetes devices (7/17, 41% overall and 7/11, 64% among diabetes clinicians).

There’s so many other things to address/time.... For example, today I feel awful...I had a kid whom I had never met, on a closed loop, and I literally could have done so much more for him...I just had no time.

Diabetes clinician #6

The biggest barrier is like just helping people troubleshoot things at the beginning... when things don’t go well at the beginning, I feel like that just leads to long term problems.

Diabetes clinician #3

#### Solution 4: Administrative Support to Offload Logistical Burden for Clinicians

Overall, diabetes clinicians agreed (6/17, 35% overall and 6/11, 55% among diabetes clinicians) that access to a specialized CHW who could provide diabetes device support would save time and improve the diabetes technology experience for both youth with T1D and their clinicians.

It is nice when we have someone with us in clinic who does have that time to spend.

Diabetes clinician #3

I think having someone who can closely follow... and then also just having time or someone to spend time going over the technology... but it doesn’t necessarily, I think, have to be a provider.

Diabetes clinician #3

Furthermore, participants (4/17, 24%) emphasized the importance of interactive and hands-on diabetes technology training to feel more confident. Two diabetes clinicians stated that wearing devices themselves greatly enhanced their learning.

## Discussion

### Principal Findings

This study presents the perspectives of diabetes and school-based clinicians on solutions that a CHW can deliver to address barriers associated with diabetes technology use among Black and Hispanic youth with T1D. Four key solutions emerged: (1) HRSN identification and navigation; (2) peer support for caregivers to navigate diabetes device logistics; (3) acting as a school liaison to improve communication between caregivers, schools, and diabetes clinicians; and (4) administrative support to offload the logistical burdens of clinicians.

Regarding HRSNs, previous literature has shown that HRSNs can interfere with diabetes technology use [33,41,42]. However, few studies explored the impact of HRSNs on diabetes device use in school settings. Our study found that school clinicians faced challenges related to both general and diabetes technology–specific social needs. School clinicians often lack the time or support to manage these HRSNs. As a result, HRSNs can disrupt the student’s education and increase the workload for school clinicians. Therefore, our participants proposed involving a CHW to perform social care navigation. Although a CHW’s core role is to provide social care navigation [43], they can serve as a bridge between schools with hospital systems to address HRSNs [44], making this a compelling solution.

In addition, the participants recognized that many caregivers struggled with the organizational skills required to manage diabetes technology, often leading to inconsistent use. They envisioned a new diabetes team member who could function as a peer mentor to empower families to perform care coordination. Previous studies have employed CHWs to encourage disease self-management using motivational interviewing techniques [45]. CHWs are ideal candidates to be health coaches or deliver motivational interviewing techniques because they are trusted community members [43]. They have the potential to positively impact youth with T1D and their caregivers to manage diabetes technology independently.

Regarding the lack of diabetes support in school, diabetes clinicians also desired someone to help caregivers advocate for their child’s rights in the school setting. Despite encouragement from the medical team, caregivers do not always seek school medical accommodations for unclear reasons. While this study did not explore the causes, possible challenges may include mistrust in the health care and education systems, language barriers, low medical literacy, and competing priorities [46,47]. Given their shared experiences with caregivers and ability to foster trust [33,43], CHWs may be more effective in motivating caregivers to seek additional school services (eg, attend medical accommodation meetings).

Regarding troubleshooting diabetes technology, both clinician types requested ongoing education, specifically more hands-on opportunities. While web-based interventions have risen in popularity for diabetes device education [48-51], our study found that clinicians strongly prefer in-person training. In addition, diabetes clinicians emphasized the importance of wearing the technology on their bodies as part of their learning process. The opportunity to wear diabetes devices should also be offered to school clinicians to increase their understanding and confidence in using diabetes technology. In addition, school clinicians desired personalized training tailored to their students’ specific devices. While most CHW interventions focus on educating patients, a study found that CHWs were open to collaborating and educating school nurses [52]. Furthermore, our findings suggest that school-based clinicians are receptive to a CHW educating them on diabetes devices, specifically their student’s devices. However, there are some reservations from both school and diabetes clinicians regarding the CHWs’ level of training in diabetes technology [53].

### Limitations

Our study has several limitations. First, the present analysis was based on the clinicians’ perspectives and did not elicit the perspectives of other school personnel, including teachers or paraprofessionals. Future efforts should include these additional stakeholders to ensure the feasibility and buy-in of the intervention across all groups. Second, participants were allowed to brainstorm freely without considering the limitations of a CHW role, which was consistent with human-centered design principles [32]. The authors recognize that it may not be possible for CHWs to perform all the solutions presented. However, allowing participants to cocreate solutions without restrictions enabled the study team to fully understand participants’ perspectives.

### Conclusions

In conclusion, this study used a novel human-centered design methodology to better understand barriers to diabetes technology use for Black and Hispanic youth with T1D from the perspectives of their diabetes and school-based clinicians. In addition, this workshop encourages CHW intervention prototyping to overcome perceived barriers. Including the perspectives of diabetes and school-based clinicians in the design process may help enhance the feasibility and acceptability of a future T1D CHW care model that aims to connect clinics and schools. The next step is to integrate the perspectives of caregivers and youth with T1D on a T1D CHW specialty care model, as their insights will further enrich the intervention development.

## Abbreviations

CGM: continuous glucose monitor
CHAM: Children’s Hospital at Montefiore Einstein
CHW: community health worker
HRSN: health-related social need
MSHP: Montefiore School Health Program
REDCap: Research Electronic Data Capture
T1D: type 1 diabetes

## Data Availability

Some or all datasets generated during or analyzed during this study are not publicly available but are available from the corresponding author on reasonable request.
